# Risks of borderline liver enzyme abnormalities to the incidence of impaired fasting glucose and diabetes mellitus: a 7 year follow up study of workers

**DOI:** 10.1186/s40557-016-0105-4

**Published:** 2016-04-07

**Authors:** Jin-Hyun Yu, Jin-Seok Kim, Mee-Ra Lee, Seong-Yong Yoon, Seong-Yong Cho, Seung-Hyun Yoo, Boo-Il Kim

**Affiliations:** Department of Occupational and Environmental Medicine, Soonchunhyang University Gumi Hospital, 179, Gongdan 1-dong, Gumi-si, Gyeongbuk, 730-706 South Korea; LIGnex1 Gumi company, 133, Gongdan 1-dong, Gumi-si, Gyeongbuk, 730-703 South Korea

**Keywords:** Gamma-glutamyltransferase, Alanine aminotransferase, Blood glucose, Diabetes mellitus

## Abstract

**Background:**

The aim of this study was to identify the relationships between borderline serum liver enzyme abnormalities and the incidence of impaired fasting glucose (IFG) and diabetes mellitus (DM) during a 7-year follow-up of workers, and to evaluate the quantitative level of risks.

**Methods:**

A total of 749 workers in an electronics manufacturing company were divided into the normal fasting blood glucose (*n* = 633), IFG (*n* = 98), and DM (*n* = 18) groups, according to the results of their health checkup in 2006. Among 633 workers in the normal group, excluding 55 workers who were impossible to follow, incidence rate and relative risks of 578 workers to the IFG or DM in 2013 according to the levels of aspartate aminotransferase (AST), alanine aminotransferase (ALT), and gamma-glutamyltransferase (γ-GTP) were investigated. The liver enzyme levels were categorized as A (normal), B (borderline elevation), and R (definite elevation) following the standard of the National Health Insurance Service of Korea.

**Results:**

The incidence rate of IFG or DM based on ALT level was 9.7 % for the A, 30.0 % for B, and 15.4 % for R. According to γ-GTP, the incidence rate was 9.8 % for A, 34.5 % for B, and 25.0 % for R. The relative risk(RR) to the incidence of IFG or DM depending on the level of ALT were 3.09 in B and 1.59 in R compared to A. According to γ-GTP, RR was 3.52 in B and 2.55 in R compared to A. AST level was not related to the incidence of IFG or DM. A multiple logistic regression analysis with the incidence of IFG or DM as a dependent variable resulted in an odds ratio of 2.664(1.214–5.849) for B level ALT, 3.685(1.405–9.667) for B level of γ-GTP even after adjustment for other variables such as age, sex, body mass index, AUDIT score, systolic blood pressure, and triglyceride.

**Conclusions:**

Even borderline elevations of ALT and γ-GTP, but not AST, increased the incidence and risk of IFG or DM after 7 years. Borderline elevation of ALT and γ-GTP was identified as an independent risk factor of IFG or DM.

## Background

Diabetes mellitus (DM) is a main risk factor of cerebro-cardiovascular diseases along with hypertension and hyperlipidemia, and its incidence tends to rapidly increase [1]. According to the report of the World Health Organization, the total number of people with DM is projected to rise from 171 million in 2000 to 366 million in 2030 [2]. In Korea, the DM prevalence rate was estimated to be <1 % of the population in the 1970s. In 2003, there were a total of 2,694,220 DM patients between 20 and 79 years old, accounting for 7.75 % of the total population, and new patients corresponding to 10 % of the total DM patients are additionally found every year [3].

Serum liver enzymes, including aspartate aminotransferase (AST), alanine aminotransferase (ALT), and gamma-glutamyltransferase (γ-GTP), are indices of liver dysfunction. In particular, γ-GTP has been used as a clinical and epidemiological index of hepatobiliary diseases and alcohol intake [4]. γ-GTP has been reported to have a strong relation with obesity and fatty liver, and to contribute to insulin resistance [5]. According to multiple previous studies, it was turned out that γ-GTP playing an important intracellular antioxidant role by maintaining a high glutathione concentration. Therefore, it was suggested to be a potential biomarker of oxidative stress [6–8]. AST, ALT and γ-GTP is three major enzyme that have been included in periodic health checkup in working population in Korea. Increases in the serum liver enzyme levels may indicative of beginning of the development of life style related disease in young workers. However, its seriousness is often not recognized because of the absence of symptoms. If when worker’s liver enzyme levels were high enough, it is fully expected that possibility of hepatobiliary diseases or other life style related disease is high. But when the abnormality level is minimal, it is usually neglected by workers themselves and also by occupational health manager. Therefore, it is necessary to accumulate scientific evidences showing that even mild increase of serum liver enzymes is a indicative signal of diseases caused by the accumulation of unhealthy life habits, and that it would progress to chronic disease such as DM unless the health related life habits are improved.

Multiple studies revealed that serum γ-GTP was an independent risk factor that could predict the incidences of type 2 DM and impaired fasting glucose (IFG) [9–12]. Elevation of γ-GTP was related to cardiovascular disease and metabolic syndrome [13, 14]. Serum ALT also was shown to be a factor that predicted DM incidence in some prospective studies [15–18], and ALT concentration was reported to be a useful index for predicting metabolic syndrome [19]. Although there have been many studies on the relationships between liver enzymes and IFG and DM, scientific evidence about amount of effect of borderline abnormality of liver enzyme is still lacking. Therefore, it is necessary to conduct a study that can directly helpful in the health management of workers, based on the abnormality of serum liver enzymes in working population. Especially, we want to know how much risks can borderline liver enzyme abnormalities increase to the occurrence of IFG or DM.

The purpose of this study was to identify the relationships between serum liver enzyme levels and the incidences of IFG and DM during a 7-year follow-up of workers, and to evaluate the quantitative level of risks in borderline elevation of liver enzyme levels.

## Methods

**Fig. 1:**
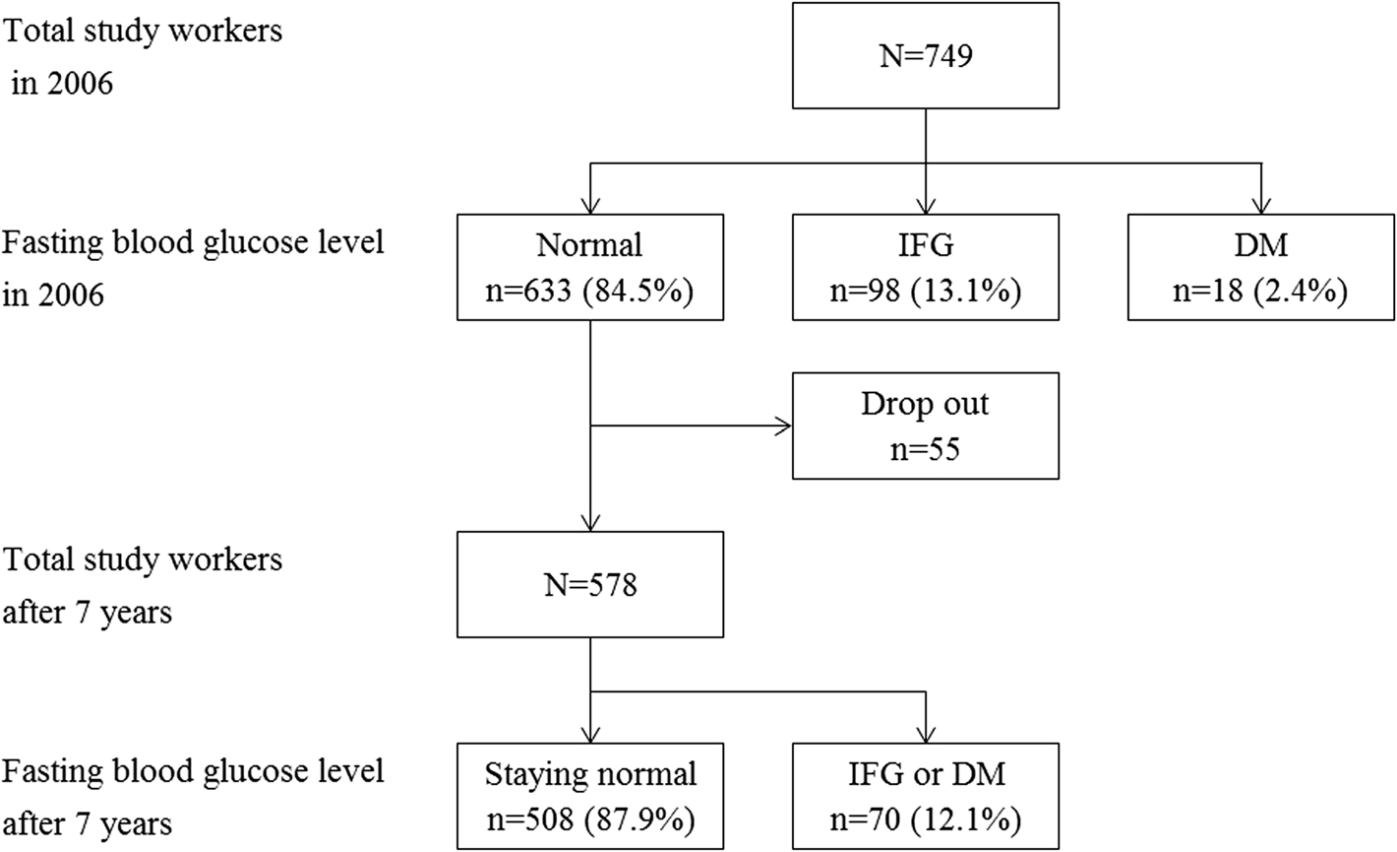
Study flow diagram. IFG, impaired fasting glucose; DM, diabetes mellitus

The research subjects were 749 workers in an electronics manufacturing company located in Korea. The workers were divided into the normal, IFG, and DM group according to their fasting blood glucose levels and present medical history based on health checkup results in 2006. There were 633(84.5 %) workers in the normal, 98(13.1 %) in the IFG, and 18(2.4 %) in the DM group. Then, among the 633 normal group workers, new development of IFG or DM based on the health checkup results in 2013 were investigated in 578 workers, excluding 55 workers who were impossible to follow because of resignation, maternity leave, or a long-term business trip (Fig. 1).

The health checkup was done following the regulation of the National Health Insurance Service (NHIS) of Korea, in the same hospital in the year of 2006 and 2013. All workers were asked to agree to the academic use of the health checkup results by filling out a consent form. Disease history and health related behaviors were surveyed by using the self-administered health risk assessment survey form used in the NHIS of Korea. Concerning smoking status, the workers were categorized as non-smokers group, ex-smokers group or current smokers group. Alcohol drinking was investigated by using the AUDIT-K [20], in which the unhealthy alcohol drinking group was defined as workers with a score of ≥10 points for males and ≥8 points for females, of a total of 40 points in AUDIT-K. The workers were also divided according to weekly number of exercise: no exercise, one to two times per week, or three times or more per week. Occupations were divided into office work, production work, and research work, and job positions were divided into executive position and general position. Blood test was performed after fasting for >8 h, in which hemoglobin, fasting blood glucose, AST, ALT, γ-GTP, total cholesterol, triglyceride, and High density lipoprotein cholesterol (HDL-C) were measured.

According to the criteria of DM diagnosis by the American Diabetes Association in 2014 [21], a normal blood glucose level was defined as a value of <100 mg/dL after 8 h of fasting, IFG as 100 to <126 mg/dL, and DM as ≥126 mg/dL. If subjects had medical history of DM, they were classified as DM even if their blood glucose level were lower than 125 mg/dL. Since there were only 4 new cases of DM in the health checkup in 2013, all workers in the IFG and the DM group were together defined as ‘IFG or DM’ as dependent variable.

The levels of serum liver enzymes were categorized as A (normal), B (borderline elevation), and R (definite elevation) following the standard of the NHIS of Korea [22]. Grade A was defined as ≤40 IU/L for AST, ≤35 IU/L for ALT, or ≤63 IU/L in males or ≤35 IU/L in females for γ-GTP. On the other hand, B was defined as 41–50 IU/L for AST, 36–45 IU/L for ALT, and 64–77 IU/L in males or 36–45 IU/L in females for γ-GTP. R was defined as ≥51 IU/L for AST, ≥46 IU/L for ALT, and ≥78 IU/L in males or ≥46 IU/L in females for γ-GTP. When serum liver enzymes were considered as dichotomous variables, the values were classified as normal for A and abnormal for both B and R.

The baseline demographic characteristics, health related behaviors, and laboratory test results of the normal, IFG, and DM group were cross-sectionally compared using the chi-square test and ANOVA test. The characteristics between the group that maintained a normal fasting blood glucose level and the group with IFG or DM after 7 years were compared by using the chi-square test and ANOVA test. The incidence rate and relative risks to IFG or DM after 7 years depending on the initial serum liver enzyme levels were calculated and compared by using the chi-square test. To analyze the effects of elevated serum liver enzymes, after the adjustment for other variables, multiple logistic regression analysis was performed. Statistical analysis was performed by using SPSS version 14 (SPSS, Inc., Chicago, IL, USA).

## Results

The characteristics of 633(84.5 %) workers in initial normal fasting blood glucose group, 98(13.1 %) in the IFG group, and 18(2.4 %) in the DM group were compared. The percentage IFG and the DM in the male was 16.9 %, which was higher than 2.8 % in the female. The workers’ age was 38.9 ± 7.95 years for the normal, 45.4 ± 7.79 years for the IFG, and 47.0 ± 8.68 years for the DM group. Length of work history was 9.2 ± 7.67 years for the normal, 15.1 ± 8.43 years for the IFG and 15.8 ± 6.41 years for DM group, showing that the group with high blood glucose levels had an older age and a longer working history. There was no difference depending on types of occupation. Executive workers had higher proportion of IFG and DM than general workers. There were no differences in alcohol drinking, smoking, exercise among the three groups. Body mass index (BMI), systolic blood pressure(BP), hemoglobin, AST, ALT, γ-GTP, total cholesterol, and triglyceride, excluding diastolic BP and HDL-C, showed unhealthier results in the group with a higher blood glucose (*p* < 0.05) (Table 1).

**Table 1:** Baseline characteristics of the study subjects according to initial serum fasting glucose level

Variables	Normal	IFG	DM	*p*-value^*^
	*n* = 633 (84.5 %)	*n* = 98 (13.1 %)	*n* = 18 (2.4 %)	
Demographic variables				
Sex^a^				0.005
Male	561 (83.1)	97 (14.4)	17 (2.5)	
Female	72 (97.3)	1 (1.4)	1 (1.4)	
Age (year)^b^	38.9 ± 7.95	45.4 ± 7.79	47.0 ± 8.68	0.000
Work duration (year)^b^	9.2 ± 7.67	15.1 ± 8.43	15.8 ± 6.41	0.000
Occupation^a^				0.056
Office work	110 (79.1)	21 (15.1)	8 (5.8)	
Production work	442 (85.8)	65 (12.6)	8 (1.6)	
Research work	81 (85.3)	12 (12.6)	2 (2.1)	
Job position^a^				0.000
Executive	159 (70.4)	56 (24.8)	11 (4.9)	
General	474 (90.6)	42 (8.0)	7 (1.3)	
Health related behaviors^a^				
Alcohol drinking				0.989
Healthy drinking	393 (84.8)	60 (12.8)	11 (2.4)	
Unhealthy drinking	235 (84.5)	36 (12.9)	7 (2.5)	
Smoking				0.647
Non-smoker	308 (84.8)	49 (13.5)	6 (1.7)	
Ex-smoker	81 (81.8)	14 (14.1)	4 (4.0)	
Current smoker	240 (84.8)	35 (12.4)	8 (2.8)	
Exercise per week				0.118
0	233 (86.9)	25 (9.3)	10 (3.7)	
1-2	265 (82.8)	49 (15.3)	6 (1.9)	
> 3	125 (85.6)	19 (13.0)	2 (1.4)	
Laboratory test results^b^				
BMI (kg/m^2^)	23.5 ± 2.95	24.6 ± 2.72	25.3 ± 2.98	0.000
Systolic BP (mmHg)	115.6 ± 11.15	123.4 ± 14.68	125.6 ± 11.74	0.000
Diastolic BP (mmHg)	70.8 ± 7.65	77.1 ± 11.39	76.3 ± 8.77	0.000
Hemoglobin (g/dL)	14.3 ± 1.37	14.6 ± 1.13	14.8 ± 1.23	0.032
AST (IU/L)	24.3 ± 8.81	25.9 ± 18.90	31.7 ± 28.47	0.015
ALT (IU/L)	26.7 ± 17.91	32.8 ± 30.00	46.4 ± 40.64	0.000
γ-GTP (IU/L)	36.2 ± 28.29	53.6 ± 45.80	67.4 ± 66.67	0.000
Total cholesterol (mg/dL)	188.9 ± 33.32	197.5 ± 36.59	221.7 ± 45.49	0.000
Triglyceride (mg/dL)	135.2 ± 98.86	162.3 ± 80.80	175.3 ± 107.62	0.018
HDL-C (mg/dL)	55.6 ± 12.37	52.0 ± 11.86	57.5 ± 15.67	0.032

*IFG* impaired fasting glucose, *DM* diabetes mellitus, *BMI* body mass index, *BP* blood pressure, *AST* aspartate aminotransferase, *ALT* alanine aminotransferase, *γ*-*GTP* Gamma-glutamyltransferase, *HDL* High density lipoprotein

^*^Chi-square test or ANOVA test

^a^N (%)

^b^Mean ± SD

The incidence of IFG or DM after 7 years in 578 initial normal workers was 12.1 % (70 workers). There were no differences in sex, age, work duration, type of occupation, job position, alcohol drinking, smoking, and exercise between normal and IFG or DM group. There were no differences in hemoglobin, AST, and total cholesterol, whereas IFG or DM group showed unhealthier results in BMI, systolic BP, diastolic BP, ALT, γ-GTP, triglyceride, and HDL-C (*p* < 0.05) (Table 2).

**Table 2:** Comparison of initial variables according to the blood glucose level change over 7-years interval

Variables	Staying normal	IFG or DM	*p*-value^*^
	(*n* = 508, 87.9 %)	(*n* = 70, 12.1 %)	
Demographic variables			
Sex^a^			0.534
Male	452 (87.6)	64 (12.4)	
Female	56 (90.3)	6 (9.7)	
Age (year)^b^	38.3 ± 7.89	39.5 ± 6.63	0.155
Work duration (year)^b^	8.7 ± 7.67	9.8 ± 6.97	0.252
Occupation^a^			0.343
Office work	87 (83.7)	17 (16.3)	
Production work	350 (88.8)	44 (11.2)	
Research work	71 (88.8)	9 (11.3)	
Job position^a^			0.925
Executive	126 (88.1)	17 (11.9)	
General	382 (87.8)	53 (12.2)	
Health related behaviors^a^			
Alcohol drinking			0.311
Healthy drinking	322 (89.0)	40 (11.0)	
Unhealthy drinking	186 (86.1)	30 (13.9)	
Smoking			0.304
Non-smoker	258 (89.9)	29 (10.1)	
Ex-smoker	63 (87.5)	9 (12.5)	
Current smoker	187 (85.4)	32 (14.6)	
Exercise per week			0.995
0	185 (87.7)	26 (12.3)	
1-2	214 (87.7)	30 (12.3)	
≥ 3	103 (88.0)	14 (12.0)	
Laboratory test results^b^			
BMI (kg/m^2^)	23.3 ± 2.83	24.9 ± 3.34	0.000
Systolic BP (mmHg)	115.0 ± 11.07	120.2 ± 11.87	0.000
Diastolic BP (mmHg)	70.6 ± 7.74	73.6 ± 7.12	0.002
Hemoglobin (g/dL)	14.3 ± 1.33	14.5 ± 1.24	0.118
AST (IU/L)	24.0 ± 8.44	26.2 ± 11.77	0.125
ALT (IU/L)	26.0 ± 17.86	32.0 ± 19.29	0.009
γ-GTP (IU/L)	34.8 ± 27.06	44.3 ± 28.78	0.006
Total cholesterol (mg/dL)	188.2 ± 33.82	194.0 ± 32.52	0.189
Triglyceride (mg/dL)	130.0 ± 90.60	156.8 ± 137.36	0.038
HDL-cholesterol (mg/dL)	56.1 ± 12.58	52.1 ± 10.47	0.015

*IFG* impaired fasting glucose, *DM* diabetes mellitus, *BMI* body mass index, *BP* blood pressure, *AST* aspartate aminotransferase, *ALT* alanine aminotransferase, *γ*-*GTP* Gamma-glutamyltransferase, *HDL* High density lipoprotein

^*^Chi-square test or ANOVA test

^a^N (%)

^b^Mean ± SD

When the incidence rate of IFG or DM after 7 years were compared according to the levels of initial serum liver enzymes, it was noted in 11.8 % with A level of AST, in 20.0 % with B, and in 18.2 % with R, which were not statistically significant different. The incidence rates of IFG or DM with A, B, and R levels of ALT resulted in 9.7, 30.0, and 15.4 %, respectively, showing significant differences. The incidence rates with A, B, and R levels of γ-GTP resulted in 9.8, 34.5, and 25.0 %, respectively, showing significant differences. When the incidence rates of IFG or DM was compared depending on the number of abnormal serum liver enzyme, 20.0 % was noted in the group with abnormal levels of one enzyme, 23.8 % with two enzymes, and 33.3 % with three enzymes, which showed that a higher number of abnormal serum liver enzymes resulted in a higher incidence rate of IFG or DM. The relative risk (RR) of ALT to the incidence of IFG or DM was 3.09 with the B, and 1.59 with the R compared to the A level of ALT. The RR of γ-GTP was 3.52 with the B, and 2.55 with the R compared to the A level of γ-GTP. The RR was 2.27 with abnormal levels of one liver enzyme, 2.70 in two enzymes, and 3.78 in three enzymes compared with the group with no abnormal levels (*p* < 0.05) (Table 3).

**Table 3:** Relative risks to the incidence of IFG or DM according to initial liver enzyme levels

Levels of initial liver enzyme	Blood glucose level		Relative Risk	*p*-value^*^
	Staying normal	IFG or DM		
AST (IU/L)				0.305
A^a^	487 (88.2)	65 (11.8)	1	
B^b^	12 (80.0)	3 (20.0)	1.69	
R^c^	9 (81.8)	2 (18.2)	1.54	
ALT (IU/L)				0.011
A	418 (90.3)	45 (9.7)	1	
B	35 (70.0)	15 (30.0)	3.09	
R	55 (84.6)	10 (15.4)	1.59	
γ-GTP (IU/L)				0.000
A	459 (90.2)	50 (9.8)	1	
B	19 (65.5)	10 (34.5)	3.52	
R	30 (75.0)	10 (25.0)	2.55	
Number of abnormal liver enzymes				0.000
0	396 (91.2)	38 (8.8)	1	
1	72 (80.0)	18 (20.0)	2.27	
2	32 (76.2)	10 (23.8)	2.70	
3	8 (66.7)	4 (33.3)	3.78	

*IFG* impaired fasting glucose, *DM* diabetes mellitus, *AST* aspartate aminotransferase, *ALT* alanine aminotransferase, *γ*-*GTP* gamma-glutamyltransferase

^a^A was defined as ≤40 IU/L for AST, ≤35 IU/L for ALT, or ≤63 IU/L in males or ≤35 IU/L in females for γ-GTP

^b^B was defined as 41–50 IU/L for AST, 36–45 IU/L for ALT, and 64–77 IU/L in males or 36–45 IU/L in females for γ-GTP

^c^R was defined as ≥51 IU/L for AST, ≥46 IU/L for ALT, and ≥78 IU/L in males or ≥46 IU/L in females for γ-GTP

^*^Chi-square test

A multiple logistic regression analysis was performed with IFG or DM incidence as a dependent variable after adjusting for the effects of other variables. For crude odds ratio(OR), B level of ALT, B level of γ-GTP and R level of γ-GTP showed significant OR of 3.981(2.020–7.846), 4.832(2.129–10.964) and 3.060(1.413–6.628) respectively. Model 1, with adjustment for age and sex, B level of ALT, B level of γ-GTP and R level of γ-GTP showed an OR of 3.967(2.002–7.860), 4.654(2.025–10.696) and 2.978(1.366–6.494) respectively, showing a significantly higher risk. Model 2 with the addition of BMI to Model 1, B level of ALT and B level of γ-GTP showed an OR of 3.002(1.471–6.127) and 3.669(1.562–8.618) respectively, showing a significantly higher risk. When risk was analyzed for Model 3 with the addition of AUDIT score, systolic BP and triglyceride to Model 2, B level of ALT and B level of γ-GTP showed an OR of 2.664(1.214–5.849) and 3.685(1.405–9.667), respectively, showing a significantly higher risk even after the adjustment for other variables, including age, sex, BMI, AUDIT score, systolic BP, and triglyceride (Table 4).

**Table 4:** Adjusted odds ratio of initial liver enzyme categories for IFG or DM

		Dependent variable = Incidence of IFG or DM			
		Crude	Model 1	Model 2	Model 3
AST	A	1	1	1	1
	B	1.873(0.515–6.814)	1.776(0.486–6.491)	1.252(0.338–4.641)	1.477(0.380–5.737)
	R	1.665(0.352–7.875)	1.685(0.355–8.001)	1.229(0.251–6.014)	1.120(0.215–5.836)
ALT	A	1	1	1	1
	B	3.981(2.020–7.846)	3.967(2.002–7.860)	3.002(1.471–6.127)	2.664(1.214–5.849)
	R	1.689(0.805–3.542)	1.653(0.784–3.484)	1.060(0.476–2.356)	1.039(0.446–2.421)
γ-GTP	A	1	1	1	1
	B	4.832(2.129–10.964)	4.654(2.025–10.696)	3.669(1.562–8.618)	3.685(1.405–9.667)
	R	3.060(1.413–6.628)	2.978(1.366–6.494)	2.021(0.888–4.598)	2.149(0.855–5.404)

*IFG* impaired fasting glucose, *DM* diabetes mellitus, *AST* aspartate aminotransferase, *ALT* alanine aminotransferase, *γ*-*GTP* gamma-glutamyltransferase

Model 1 adjusted for age, sex

Model 2 adjusted for age, sex, body mass index

Model 3 adjusted for age, sex, body mass index, AUDIT score, systolic blood pressure, triglyceride

Odds ratio and 95 % confidence interval by multiple logistic regression analysis

## Discussion

Among three serum liver enzymes, AST had no effect on the incidence of IFG or DM after 7 years. Our results are consistent with those reported by Nakanishi et al. who found no association of AST with diabetes risk after adjustment for other known risk factors in a study of male Japanese office workers [23]. The groups with elevated ALT and γ-GTP had high risks of IFG or DM incidence. These findings are consistent with results of previous studies on the relations of ALT and γ-GTP with the incidences of IFG or DM. In a 2-year prospective study by Cho et al., serum ALT was a factor predicting the incidence of DM after adjustment for age, family history, BMI, amount of alcohol drinking, and insulin resistance [17]. Ohlson et al. reported that baseline ALT was a predictor of the incidence of type 2 diabetes after 13.5 years of follow-up in a cohort of 766 Swedish males, with a significant fourfold increased risk for males in the upper quintile compared to the lowest quintile [24]. Vozarova et al. found that higher ALT was a significant and independent predictor of incident type 2 diabetes mellitus with a two-fold increased risk, after adjustment for age, sex, body fat, insulin sensitivity and acute insulin response [18]. Although the mechanism underlying the associations between ALT and incidence of DM remains unclear, some possibilities can be considered. One is that increased serum ALT levels reflect an excess deposit of fat in the liver, a condition known as non-alcoholic fatty liver disease (NAFLD) [25]. NAFLD, which is closely related to obesity and visceral fat deposition, is now regarded as a feature of insulin resistance syndrome [5], and visceral adipose tissue is known to confer a significantly higher risk of type 2 diabetes [26]. Excess visceral fat accumulation may be causally related to features of insulin resistance as well as to high plasma levels of insulin and glucose [27, 28].

Our study findings are also consistent with results of studies on the relations between γ-GTP and the incidences of IFG and type 2 DM [13, 29, 30], and also consistent with a study showing that γ-GTP, as an index of oxidative stress, was an independent risk factor of cerebro-cardiovascular diseases [31]. Although the mechanism to explain the relationship of γ-GTP with DM was not fully elucidated, a number of previous studies have proposed some hypotheses as follows. First, serum γ-GTP is an index of insulin resistance related to non-alcoholic fatty liver, and elevation of serum γ-GTP is the result of obesity and deposition of visceral fat [5, 32, 33]. Second, elevation of γ-GTP results in chronic inflammation, which, in turn, inhibits insulin metabolism in other organs as well as in the liver, and is involved in the production of cytokines such as TNF-α and IL-6 that are involved in fatty liver formation [34–36]. Third, increased γ-GTP concentration in the blood may be a response to oxidative stress in the body, and oxidative stress plays an important role in the mechanism of DM development [7, 37].

As we analyzed the relationships between liver enzyme levels and incidence of IFG or DM, we can see characteristic results about borderline elevation of liver enzyme levels. We categorized the levels of serum liver enzymes into grades A (normal), B (borderline elevation), and R (definite elevation), and found that the risks of IFG or DM for all three liver enzymes were higher in the B group than in the R group. Also, it was observed that borderline elevation of ALT and γ-GTP was an independent risk factor of development of IFG or DM, showing a significant result after adjustment for other variables. There were several study results concerning about dose-dependent relationships between serum γ-GTP and IFG and DM. Most of them showed a linear increase in the relation between γ-GTP and IFG. A cross-sectional study on the prevalence rates of DM and IFG depending on γ-GTP, on 8666 healthy Koreans, found that as γ-GTP increased, the risks of DM and IFG also linearly increased [38]. Another cross-sectional study involving about 29,959 healthy Koreans who visited health checkup center, when γ-GTP levels were divided into five groups within normal concentration range, γ-GTP was shown to have strong linear correlations with IFG and DM in both males and females [39]. In a 4-year follow-up study with 4088 healthy male workers, a normal range of γ-GTP showed a strong dose-response relationship with DM incidence [11]. Results of last two study suggested that an increase in γ-GTP concentration within its physiological range is a sensitive and early biomarker for the development of diabetes which consistent with our results.

There are two possible explanations for the reasons why the borderline elevation of liver enzyme had higher risk than definite elevation. First explanation is possibility of selective intervention only for R grade group. Since this study was designed as a follow-up study, workers who had determined to be R grade liver enzyme level in the initial health checkup might had recognized their own health conditions more seriously than workers with B grade. Therefore, it was possible that they could have been more active in exerting effort in managing their health and changing health related life styles. In most cases of workplace settings, active health intervention efforts of occupational health managers were targeted only for workers with R grade. Therefore, it was possible that more active interventions were likely to be conducted for those with R than B grade. It was assumed that this selective interventions play a role to leading a relatively lower risk of IFG or DM in the R than B group. Another possible explanation is that borderline elevation of liver enzymes could indicate a genuine higher risk of IFG or DM than definite elevation. We should not conclusively accept this assumption because we currently do not have sufficient evidence to support this it. However, from the point of view of prediction of future occurrence of IFG or DM, we speculate that borderline elevation of liver enzymes may indicate a higher risk than definite elevation; at the very least, the risk of IFG or DM is as high as for a definite elevation. A high rise in liver enzyme level could be usual expression of intrinsic liver pathology or index of heavy alcohol drinking rather than early predictive index of cardiovascular diseases. As for a index of cardiovascular risk factor in healthy young workers, it could possible that liver enzyme tend to be expressed as a mild elevation rather than severe elevation. Even though this assumption is lack of scientific evidence, this issue should be investigated in future more scientific research.

The limitations of the present study are as follows: First, as this study was conducted only for healthy workers, and the study sample was small, it is difficult to generalize the study results to the entire working population. The DM prevalence rate of this study was 2.4 % at initially, and that of IFG was 13.1 %. In comparison with the 10.1 % DM prevalence rate and 19.9 % IFG prevalence rate in Korea in 2010 [40], the prevalence rate of DM and IFG were relatively lower, which is speculated to be because the workers of this study were relatively younger workers who are in a good health condition. Second, as only four workers were found to have DM in the health checkup after 7 years, the abnormal blood glucose group was defined together as IFG or DM as a dependent variable. Third, because there were many missing survey questions about disease history or current health condition due to the nature of the self-administered questionnaire, disease history (e.g., hepatitis B) and medication history, family history of DM that could serve as confounding variables could not be reflected accurately. In addition, although fasting insulin level and waist circumference could serve as important confounding variables, they were not evaluated. A few studies reported that waist circumference is more useful than BMI in the prediction of DM incidence [41, 42].

Despite of few limitations, as a 7 year follow-up study, its follow-up rate was as high as 91.3 %, which demonstrates that it was appropriately designed to investigate the causal relationships between IFG or DM and serum liver enzyme levels. Since, the ranges of serum liver enzymes were divided according to the standard of the NHIS of Korea that is currently conducted on worker’s health checkup, the obtained results could be directly applicable to occupational health management. The results showing that borderline ALT or γ-GTP levels had a three times higher risk of IFG or DM after 7 years than normal workers, and that as the number of abnormal liver enzymes increased the risk also increased, can be directly used for the interpretation of health checkup results. Mild rise in liver enzyme in health checkup is likely to be a warning sign of accumulation of unhealthy life style, although young workers tend to ignore such results. On the basis of the results of this study, workers should be reminded that even a mild initial abnormality in liver enzyme levels could lead to the occurrence of chronic diseases such as IFG and DM in a few years.

## Conclusion

In conclusion, our data showed that borderline elevations of ALT and γ-GTP, but not AST, increased the incidence rate and risks of IFG or DM after 7 years, and borderline elevation of ALT and γ-GTP was an independent risk factor for the incidence of IFG or DM. The results of this study could be used as a basis for more active and detailed health management and intervention measures for workers who have borderline levels of serum liver enzymes in occupational health checkup results.

### Consent

Written informed consent was obtained from the participants for the publication of this article and any accompanying images.

## Abbreviations

IFG: Impaired fasting glucose
DM: Diabetes mellitus
AST: Aspartate aminotransferase
ALT: Alanine aminotransferase
γ-GTP: Gamma-glutamyltransferase
HTN: Hypertension
NHIS: National Health Insurance Service
AUDIT-K: Alcohol Use Disorders Identification Test-Korean revised version
HDL-C: High density lipoprotein cholesterol
BMI: Body mass index
BP: Blood pressure
RR: Relative risk
OR: Odds ratio
NAFLD: Non-alcoholic fatty liver disease
